# Optimization of an Optical Inspection System Based on the Taguchi Method for Quantitative Analysis of Point-of-Care Testing

**DOI:** 10.3390/s140916148

**Published:** 2014-09-01

**Authors:** Chia-Hsien Yeh, Zi-Qi Zhao, Pi-Lan Shen, Yu-Cheng Lin

**Affiliations:** 1 Department of Engineering Science, National Cheng Kung University, Tainan 701, Taiwan; E-Mails: chyeh@mail.ncku.edu.tw (C.-H.Y.); balaboo4561973@hotmail.com (Z.-Q.Z.); 2 Firstep Bioresearch, Incorporation, Tainan 701, Taiwan; E-Mail: rebbeca@firstep.com.tw

**Keywords:** optical inspection system, Taguchi method, detection limit, linearity

## Abstract

This study presents an optical inspection system for detecting a commercial point-of-care testing product and a new detection model covering from qualitative to quantitative analysis. Human chorionic gonadotropin (hCG) strips (cut-off value of the hCG commercial product is 25 mIU/mL) were the detection target in our study. We used a complementary metal-oxide semiconductor (CMOS) sensor to detect the colors of the test line and control line in the specific strips and to reduce the observation errors by the naked eye. To achieve better linearity between the grayscale and the concentration, and to decrease the standard deviation (increase the signal to noise ratio, S/N), the Taguchi method was used to find the optimal parameters for the optical inspection system. The pregnancy test used the principles of the lateral flow immunoassay, and the colors of the test and control line were caused by the gold nanoparticles. Because of the sandwich immunoassay model, the color of the gold nanoparticles in the test line was darkened by increasing the hCG concentration. As the results reveal, the S/N increased from 43.48 dB to 53.38 dB, and the hCG concentration detection increased from 6.25 to 50 mIU/mL with a standard deviation of less than 10%. With the optimal parameters to decrease the detection limit and to increase the linearity determined by the Taguchi method, the optical inspection system can be applied to various commercial rapid tests for the detection of ketamine, troponin I, and fatty acid binding protein (FABP).

## 1. Introduction

Human chorionic gonadotropin (hCG) is an important marker indicating pregnancy. In molecular biology, hCG is a hormone produced by the syncytiotrophoblast, a component of the fertilized egg after conception. In the early detection method, a bioassay method involving the germination of wheat by the pregnant woman's urine, or the ovulation of mice and frogs caused by implanting the pregnant woman's urine [1] was used to determine whether a woman was pregnant. In 1960, the radioimmunoassay (RIA) method was used to detect the hCG concentration [2].

In general, commercial detection reagents (hCG, ketamine, troponin I, luteinizing hormone, *etc.*) were used in the lateral flow immunoassay to detect the specific antigen, and the different color in the test line was observed by the naked eye. The pregnancy test strip was used to detect the hCG concentration in the woman's urine. The cut-off value of the pregnancy test strip was set to 25 mIU/mL. Because the pregnancy test strip was based on the immune-colloidal gold technique and the sandwich immunoassay, the color of the test line darkened when the levels of hCG concentration increased [3]. Lateral flow immunoassay had the advantages of simple operation, low cost, and rapid detection. However, the color of test line was lighter under lower levels of hCG concentration. The results of the test line were compared with the standard chroma card [4–6]. The color difference between the test line and standard chroma card was not clearly determined at lower levels by the naked eye, and the color discrimination was affected by the external environment and human factors.

Many studies have used optical sensors to capture images of the reaction area and applied image processing to quantitative analysis. In 2002, Zhang *et al.* used a charge-couple device (CCD) and digital signal processing (DSP) to perform mathematical calculations for RGB color models [7]. In 2005, Zhou *et al.* developed auto-control white balance algorithms for digital cameras [8]. In 2010, Zheng *et al.* applied image processing to biomedical detection [9]. The image capture system included a metal-oxide-semiconductor (CMOS), an enlarger lens, and a light-emitting diode (LED), and used the field programmable gate array (FPGA) and DSP to control the image capture and to perform image processing. In 2011, Jiang *et al.* utilized fuzzy theory to establish an algorithm for concentration classification [10]. Our optical inspection system used the CMOS sensor to capture the image of the reaction area (test line and control line) on the pregnancy test strip and then used grayscale processing of the test line to do quantitative analysis.

The purpose of our study was to develop a new optical inspection system to upgrade the detection method of the universal test strip from the conventional qualitative analysis to quantitative analysis. For quantitative analysis, a standard calibration curve of high linearity and low standard deviation at specific levels of concentration must be established. The Taguchi method is a statistical way to improve the quality of product performance and is also applied to engineering [11–15], biotechnology [16], marking and advertising [17–21]. Rosa *et al.* used the Taguchi method to improve the electrolytic conditions and increased the electroforming efficiency to 95% [22]. Rao *et al.* optimized the stirring conditions and inoculum dose for xylitol production, and the productivity was increased to 78.9% [23]. The CMOS sensor in our optical inspection system had ten parameters that affect the quality of the captured image, such as the black light compensation, brightness, contrast, exposure, gain, gamma, hue, saturation, sharpness value, and white balance. To increase the S/N value and to obtain a linear slope, we used the Taguchi method to find the optimal parameters for the optical inspection machine. After the optimal parameters were found, the optical inspection system can obtain a higher S/N value, lower standard deviation, more linear calibration curve (R square close to one), and a lower concentration detection limit.

## 2. Materials and Methods

### 2.1. Experimental Principles

**Figure 1.**
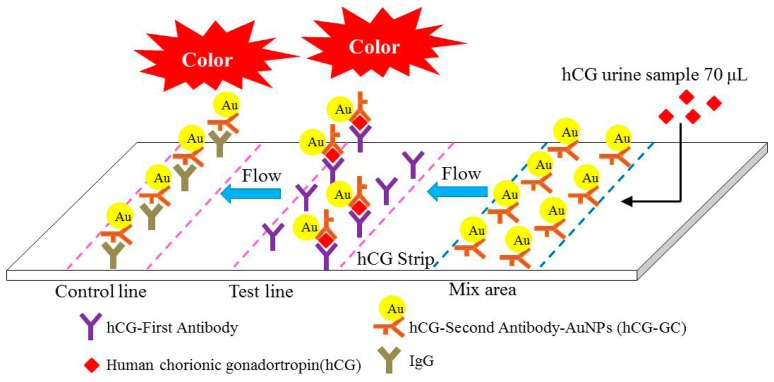
The diagram of hCG reaction method in lateral flow immunoassay.

The pregnancy test used a lateral flow sandwich immunoassay, and the pregnancy test strip included the backing card, sample pad, glass fiber, nitrocellulose membrane, and absorption pad. The anti-hCG antibody and immunoglobulin G antibody defined as the test line and control line were immobilized on the nitrocellulose membrane. The anti-hCG antibody-colloidal gold conjugates were immobilized on the glass fiber, which was defined as the mixture area. After the sample solution including the hCG antigen in the urine was dropped into the sample pad, the hCG antigen first bonded with the anti-hCG antibody-colloidal gold conjugates and then bonded with the anti-hCG antibody, as shown in Figure 1.

When the hCG concentration was increased, larger volumes of the colloid gold were aggregated in the test line and this deepens the color of the test line. Then, the optical inspection system was used to detect the color signals of the various hCG concentrations in the pregnancy test strip, and the laboratory virtual instrumentation engineering workbench (LABVIEW™) was used to control the CMOS sensor and analyze the image of the test line and control line. The CMOS sensor was a two-million-pixel camera module and supported image capture and video streaming. The CMOS sensor has the advantages of easy operation (USB 2.0), small dimensions (6 × 0.8 × 0.4 cm), and low power (input voltage 5 V). Under the grayscale analysis, the relationship between the hCG concentration and grayscale was established and the detection method of pregnancy test strip was converted from qualitative to quantitative analysis. To increase the linearity and decrease the standard deviation, we used the Taguchi method to find the suitable parameters for the CMOS sensor.

### 2.2. Design and Fabrication of the Optical Inspection Machine

The optical inspection machine, which included a CMOS sensor carrier, a set of LED lights, and a test strip carrier, was designed using AutoCAD^®^2010 software. The dimension of the optical inspection machine was 18 × 18 × 23 cm, the CMOS sensor carrier was 14 × 15 × 18 cm, and the test strip carrier was 12 × 7 × 1.2 cm. First, the case of the optical inspection machine was designed in a darkroom to protect the CMOS sensor from external light. Second, the CMOS sensor was fastened to the CMOS sensor carrier in the specific position to capture the clear image of the reaction region. Finally, the test strip carrier was used to confirm the reaction region of the test strip with the captured image of the CMOS sensor. The fabrication of the every pattern was constructed on a conventional polymethyl methacrylate (PMMA) substrate with a laser micromachining process by a CO_2_ laser machine (LaserPro Venus, GCC, New Taipei City, Taiwan).

### 2.3. Experimental Procedure

**Figure 2.**
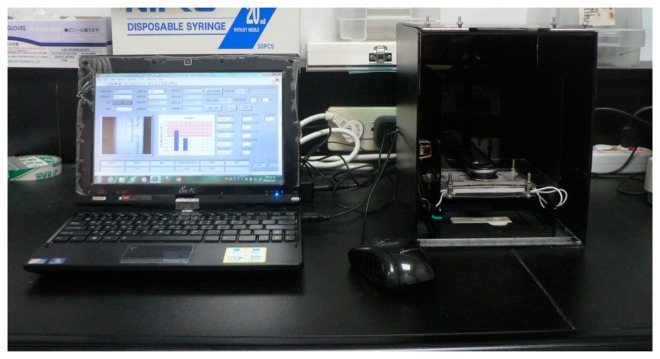
Illustration of the optical inspection system.

**Figure 3.**
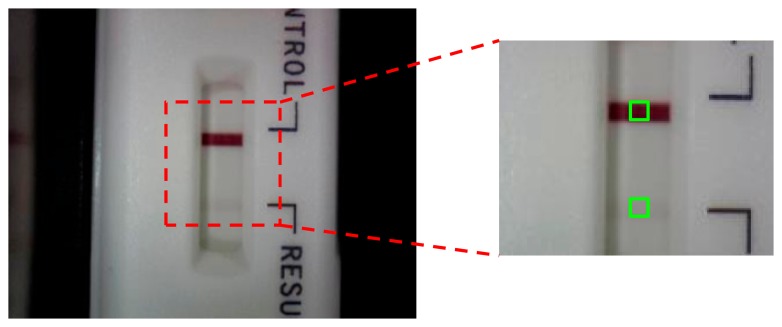
The control line and test line of the hCG strip was captured by the optical inspection system.

The model of the pregnancy test strip was a sandwich immunoassay. When the hCG concentration was increased, the color of the test line darkened. For quantitative analysis and error reduction, the optical inspection system was used to measure the color signals of the test line, as shown in Figure 2. First, to establish the optimal parameters for the optical inspection system under the lower hCG concentration conditions, we adjusted the grayscale of the standard color level from 255 to 247 to be the test line, and then the colors of the test and control line were calculated as grayscale signals, as shown in Figure 3. Second, after the best parameters were found, urine samples with various hCG concentrations (50, 37.5, 35, 12.5, and 6.25 mIU/mL) were injected into the pregnancy test strip to establish the standard calibration curve. The urine samples were prepared by the high hCG concentration (1000 mIU/mL) and blank urine purchased from Firstep Bioresearch Inc. (Tainan, Taiwan) and stored at 4 °C.

To obtain the best performance of the optical inspection system, we introduced the Taguchi method in a ten factor, two-level, L_12_ (2^11^) orthogonal array to evaluate the related optimal values. Ten typically influential parameters included the black light compensation (control factor A), brightness (control factor B), contrast (control factor C), exposure (control factor D), gain (control factor E), gamma (control factor F), hue (control factor G), saturation (control factor H), sharpness value (control factor I), and white balance (control factor J). Table 1 presents the studied control factors with two levels. The grayscale was proportion to hCG concentration. The relationship between the hCG concentration and grayscale was illustrated by the straight line, and we used the dynamic quality characteristic. The S/N function was:

(1) $$\text{S}/N=-10\text{log}\left(\frac{S_{d}^{2}}{\beta^{2}}\right)$$

where β is linear slope, S_d_ is standard deviation. The S/N was used as a larger-the-better quality characteristic. The linear slope (β) was between the measured grayscale and standard grayscale, and the ideal slope was close to one. The standard grayscales were 247, 249, 251, 253, and 255. After the optimal parameters were found, the different hCG concentrations were used to detect the limit and create a higher linear curve between the grayscale and hCG concentration with the smaller standard deviation. Finally, the optical inspection system used the specific hCG concentration to detect the grayscale and utilized the linear curve to transform the hCG concentration.

## 3. Results and Discussion

### 3.1. The Taguchi Approach

**Figure 4.**
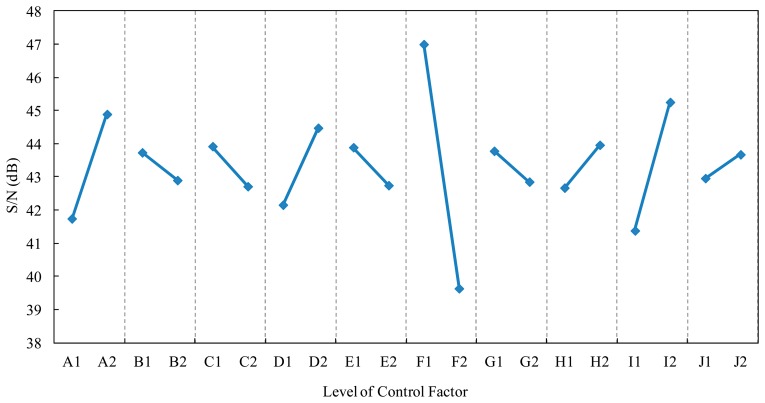
Response graph of the control factors on the S/N functions.

After the experiments were designed by the Taguchi method (L_12_ (2^11^) orthogonal array), the best levels for the factors could be determined through calculation and tabulation. Table 2 presents the influences of the control factors on the S/N function. When the control factor A transformed from level 1 to level 2, the S/N value increased from 41.74 to 44.89, and then the maximum effect of the control factor A was 3.15 dB. When the gap between the two levels of the control factor was wider, this control factor could affect the S/N value, and the control factor with higher level of S/N value was the optimal parameter. According to the S/N response graph (Figure 4), the recommended optimal levels of the control factors based on the larger-the-better S/N function were A2, B1, C1, D2, E1, F1, G1, H2, I2, J2.

In Table 2, the control factors A, F, and I had response greater than 3 dB, and these control factors (A, F, and I) affected the linearity of the dynamic results and standard deviation. To verify the optimal parameters and to eliminate the experimental errors with those control factors, we used the analysis of variance (ANOVA) to find the real control factor. In the ANOVA, when the confidence level of control factor was higher than 95%, this control factor has enough influence on the S/N value. The results reveal that the confidence levels of the control factors A, F, and I were higher than 95%, so these control factors could affect the S/N value. We only adjusted the control factor A, F, and I to optimal condition. The original levels of the control factors before the Taguchi method were A1, B1, C1, D1, E1, F1, G1, H1, I1, J1. According to the S/N response graph and ANOVA, the optimal levels of the control factors based on the larger-the-better S/N function were A2, B1, C1, D1, E1, F1, G1, H1, I2, J1.

We used the original and the optimal parameters to repeat confirmation experiments five times. The S/N ratios were based on Equation (1). After the confirmation experiments for both the original and the optimal parameters, the S/N value increased from the original experiment (43.48 dB) to the optimal experimental value (50.5 dB), which represented an increase of 7.02 dB. Moreover, when the original conditions were changed to the optimal conditions, the linear slope (β) between the measured grayscale and standard grayscale decreased from 1.0037 to 1.0004, and the standard deviation decreased from 0.0063 to 0.0035 (data not shown). The decreases indicated that the optimal parameters for the optical inspection machine had better linear slope and lower standard deviation.

**Figure 5.**
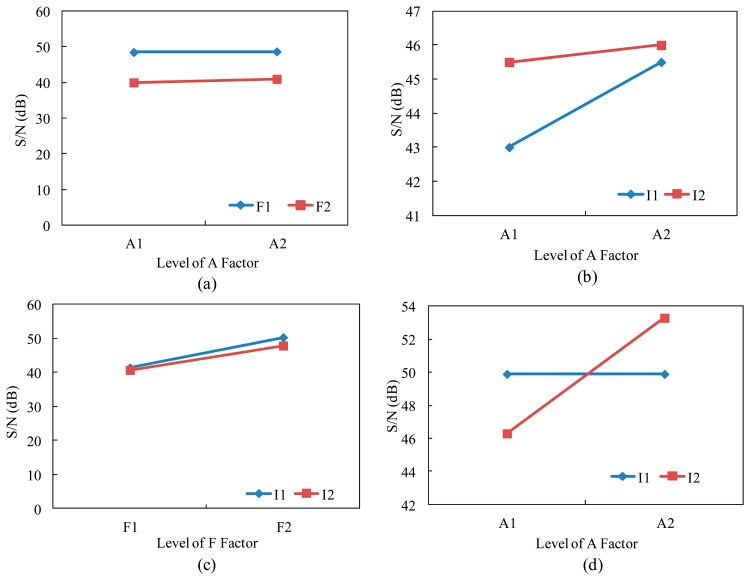
Interaction graphs of the first interaction. (**a**) A×F; (**b**) A×I; and (**c**) F×I; (**d**) The second interaction of A×I.

According to the previous experiments, control factors A, F, and I had the major effects on the S/N value. Then, the interaction relationships between the A×F, A×I, and F×I were studied. First, we finished the interaction graph of interaction A×F, A×I, and F×I, as shown in Figure 5a–c. The results of these interactions reveal that A×F and F×I had no interaction, and the maximum of S/N value occurred at A2×F1 and F1×I1. However, a strong interaction was observed between control factor A and control factor I, and we found that when the control factor A was under the level 1 and level 2, the S/N value was increased by increasing the control factor I from level 1 (sharpness value 2) to level 2 (sharpness value 3). To obtain better S/N value, we finished the second interaction graph of the A×I interaction. The level 1 and level 2 of the control factor A were TRUE and FALSE, and the level 1 and level 2 of the control factor I were sharpness value 3 and sharpness value 7. The results reveal that the interaction A×I had a strong effect (same as that of the first interaction experiment). When the control factor A was changed from level 1 to level 2, the S/N value increased from 49.91 dB to 53.38 dB with the increasing sharpness value from 3 to 7, as shown in Figure 5d. Therefore, the optimal parameters of the optical inspection system were: A2, B1, C1, D1, E1, F1, G1, H1, I2, J1. After the confirmation experiment for the original and second optimal parameters, the S/N of second optimal experimental parameters increased 9.9 dB (from 43.48 dB to 53.38 dB).

### 3.2. The hCG Concentration Test by the Optical Inspection System

**Figure 6.**
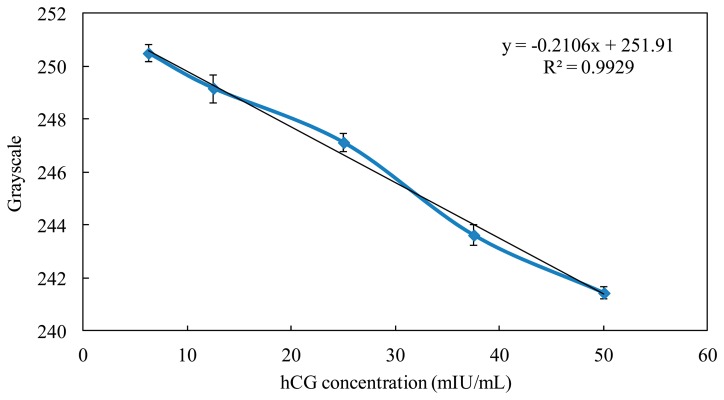
The relationship between grayscale and the hCG concentration.

After using the Taguchi method to find the optimal parameters for the optical inspection system, we used urine samples with different hCG concentrations (50, 37.5, 25, 12.5, and 6.25 mIU/mL) to establish the hCG standard calibration curve and to detect the hCG concentration limit. In the conventional qualitative detection for the hCG strips, the cut-off value was 25 mIU/mL. When the hCG concentration ranged from 6.25 to 50 mIU/mL, the calibration curve between the grayscale and hCG concentration had high linearity (R square was 0.99), and the standard deviation of every hCG concentration was less than 10%, as shown in Figure 6. Moreover, the hCG concentration detection limit was reduced to 6.25 mIU/mL by using the optical inspection system. The commercial product of the pregnancy test strip was only determined at a cut-off value of 25 mIU/mL by the naked eye, so we have successfully established the hCG standard calibration curve and improved the conventional detection method from qualitative detection to quantitative detection. Finally, we used urine samples with five standard hCG concentrations (50, 37.5, 25, 12.5, and 6.25 mIU/mL) to proceed with the hCG strip detection by the optical inspection system. After the grayscale signals were transformed to hCG concentration by the calibration curve, each concentration error was less than 10%. Therefore, the optical inspection system can detect the unknown hCG concentration in the strip using the calibration curve.

## 4. Conclusions

We have successfully demonstrated the optimization of an optical inspection system for hCG concentration detection by using the Taguchi method. The results reveal that S/N value increased to 53.38 dB, and the linear slope was close to that obtained using the optimal parameters. The optical inspection system established a highly linear calibration curve for hCG concentration, and the detection error of the hCG concentration was less than 10%. The conventional qualitative detection method for any test strip was upgraded to quantitative detection, and the detection limit of specific concentration was below the traditional cut-off value. In the future, the newly optical inspection system could be applied to other clinical strip detection and color tests.

## Figures and Tables

**Table 1.** The experiential control factors for the Taguchi approach.

Control Factors		Level	

		1	2
A	Backlight compensation	TRUE	FALSE
B	Brightness	−8	−4
C	Contrast	0.4	0.5
D	Exposure	0.4	0.3
E	Gain	2	0
F	Gamma	1	3
G	Hue	0	0.4
H	Saturation	50	100
I	Sharpness	0	3
J	White balance	Auto	2800

**Table 2.** Effects of control factors on the S/N functions.

**Factor**	**Level 1**	**Level 2**	**E^1-2^**	**Range**	**Rank**
A	41.7435	44.8924	3.1489	3.1489	3
B	43.7364	42.8995	−0.8368	0.8368	9
C	43.9219	42.7140	−1.2080	1.2080	6
D	42.1578	44.4781	2.3204	2.3204	4
E	43.8906	42.7453	−1.1452	1.1452	7
F	46.9984	39.6375	−7.3609	7.3609	1
G	43.7826	42.8533	−0.9292	0.9292	8
H	42.6729	43.9630	1.2902	1.2902	5
I	41.3817	45.2542	3.8725	3.8725	2
J	42.9600	43.6759	0.7160	0.7160	10
K	43.5897	43.0462	−0.5435	0.5435	11
